# Follicle-stimulating hormone induces depression-like phenotype by affecting synaptic function

**DOI:** 10.3389/fnmol.2024.1459858

**Published:** 2024-10-21

**Authors:** Liqin Huang, Shangqi Sun, Gege Jiang, Guanfeng Xie, Yunying Yang, Sichun Chen, Jiaying Luo, Chen Lv, Xiang Li, Jianming Liao, Zhihao Wang, Zhaohui Zhang, Jing Xiong

**Affiliations:** ^1^Department of Neurology, Renmin Hospital of Wuhan University, Wuhan, China; ^2^Department of Neurology, Tongji Medical College, Union Hospital, Huazhong University of Science of Technology, Wuhan, China; ^3^Department of Neurosurgery, Renmin Hospital of Wuhan University, Wuhan, China; ^4^Taikang Center for Life and Medical Sciences, Wuhan University, Wuhan, China

**Keywords:** depression, FSH, inflammation, synaptic plasticity, GABA, glutamate

## Abstract

Depression is one of the most common affective disorders in people’s life. Women are susceptibility to depression during puberty, peripartum and menopause transition, when they are suffering from sex hormone fluctuation. A lot of studies have demonstrated the neuroprotective effect of estrogen on depression in women, however, the effect of FSH on depression is unclear. In this study, we investigated the role of FSH on depression in mice. Our study demonstrated that FSH induced depression-like behaviors in mice in a dose-dependent manner. This induction was associated with elevated levels of pro-inflammatory cytokines, including IL-1β, IL-6, and TNF-*α* in both serum and hippocampal tissues. Additionally, FSH treatment resulted in impaired synaptic plasticity and a reduction in the expression of key synaptic proteins. It is noteworthy that the depression-like behaviors, inflammatory cytokines expression and synaptic plasticity impairment induced by FSH could be alleviated by knocking down the expression of FSH receptor (FSHR) in the hippocampus of the mice. Therefore, our findings reveal that FSH may play an important role in the pathogenesis of depression and targeting FSH may be a potential therapeutic strategy for depression during hormone fluctuation in women.

## 1 Introduction

Depression is a common medical condition characterized by significant distress, morbidity, and mortality, which may diminish psychosocial functioning and reduce the quality of life (Malhi and Mann, 2018). The severity of depression ranges from depressive mood to major depressive disorder (MDD). Women are twice as likely to suffer from depression across their life time as men, particularly during periods of sex hormone fluctuation such as puberty, peripartum and menopause transition (Kuehner, 2017). Although the reasons for women’s susceptibility to depression remain unclear, a series of studies suggest that alterations in hormonal and endocrine function may play an essential role in the pathophysiological mechanism of depression (Gordon et al., 2015). Thus, exploring the effects of sex hormones on depression could provide prevention or therapeutic approaches for depression in female. The neuroprotective effects of estrogen on depression in women have been studied in several studies, but the relationship between estrogen deficiency and depression remains controversial. Researchers from the Melbourne Midlife Women’s Health Project (MMWHP) found that decreased estradiol levels were linked to symptoms of depression in a cohort of women predominantly studied during the perimenopausal period (Ryan et al., 2009). However, researchers found that testosterone levels and their fluctuations during the menopausal transition were associated with depressive symptoms, while no significant correlations were found between levels of estradiol and their changes with depressive symptoms or major depressive disorder in the Study of Women’s Health Across the Nation (SWAN) (Bromberger et al., 2007). Moreover, the clinical trials found that transdermal estradiol improved depressive symptoms in perimenopausal women, but not in postmenopausal women (Joffe et al., 2011; Cohen et al., 2003; Morrison et al., 2004). These studies indicated that fluctuations in the other hormones during perimenopause may play a role in the development of depression. Therefore, additional research is needed to explore the potential impact of these hormonal changes on women’s vulnerability to depression.

Follicle-stimulating hormone (FSH), a gonadotropin secreted by the gonadotropic cells of the anterior pituitary gland, is essential for the regulation of reproductive function in both male and female individuals (Coss, 2020). In females, fluctuations in FSH levels occur at various stages of their reproductive lifespan. During the menstrual cycle, FSH levels increase to facilitate the development and maturation of ovarian follicles, ultimately leading to the production of estrogen. FSH levels decline after ovulation, which is regulated by negative feedback from estrogen. The early menopausal transition is characterized by a significant rise in serum levels of FSH, even with normal estrogen levels. Additionally, abnormally elevated FSH levels may be indicative of conditions such as primary ovarian insufficiency (POI), pituitary tumors, or Turner syndrome (Bhartiya and Patel, 2021). FSH binds to and activates the FSHR, initiating a series of biochemical signaling pathways. Recent research has identified the expression of FSHR in extragonadal tissues, including osteoclasts, adipose tissue, hepatocytes, hippocampal and cortical neurons (Lizneva et al., 2019). The effects of FSH other than fertility have attracted much attention, such as osteoporosis (Sun et al., 2006), obesity (Liu et al., 2017), dementia (Xiong et al., 2022), and cardiovascular disorders (Guo et al., 2019) in postmenopausal female disease. However, there are limited studies on the association between FSH and depression. Various epidemiological studies have explored the link between FSH and depression, yielding contradictory findings. For instance, the multi-center multi-ethnic cohort SWAN study found no significant association between depressive symptoms and FSH levels during menopausal transition (Bromberger et al., 2011). However, in a longitudinal study involving women without a prior history of depression, researchers observed that elevated FSH levels were linked to lower rates of depression in postmenopausal women (Freeman et al., 2006; Dennerstein et al., 2004). Conversely, the MMWHP investigators discovered that significantly elevated FSH levels were correlated with the development of depressive symptoms during the menopausal transition (Ryan et al., 2009). Besides the role of FSH during menopausal transition, elevated FSH levels have been associated with an increased risk for developing depression during post-partum (Ramachandran Pillai et al., 2017). These conflicting findings have prompted further investigation into the potential role of FSH in the development of depression.

In this study, we use recombinant human FSH to stimulate male C57BL mice. We found FSH induced depressive symptoms and pathological changes in hippocampus of the mice in a dose-dependent manner. Furthermore, targeted suppression of FSHR expression in the hippocampus effectively mitigated the depressive effects induced by FSH. These findings suggest a potential significant involvement of FSH in the pathogenesis of depression.

## 2 Materials and methods

### 2.1 Materials

Recombinant human FSH protein (hor-249) was from Prospecbio; antibody to IL-6 (YT5348) was from Immuneway; antibodies to IL-1*β* (16806-1-AP), GluR1 (67642-1-Ig), FSHR (22665-1-AP), GAD67 (10408-1-AP), VGluT1 (21829-1-AP), and synaptophysin (17785-1-AP) were from Proteintech; antibodies to GluR2 (A11316), synapsin (WX620330), PSD95 (A7889), ERK1/2 (A4782), and phospho-ERK1/2 (AP0472) were from Abclonal; antibody to GFAP (AB-2532994) was from Invitrogen; antibody to Iba1 (019-19741) was from Wako; antibody to β-actin (GB11001-100) was from Servicebio; ERK1/2 inhibitor (HY-112287) was from MedChemExpress; IL-1β mouse ELISA kit (MU30369), IL-6 mouse ELISA kit (MU30044), and TNF-*α* mouse ELISA kit (MU30030) were from Bioswamp; human FSH ELISA kit (CSB-E06867h) and luteinizing hormone (LH) ELISA kit (CSB-E12770m) were from Cusabio; 17β-estradiol ELISA kit (ab108667) was from Abcam; 4′,6-diamidino-2-phenylindole (DAPI, D9542) was from Sigma-Aldrich; AAV9 sh-FSHR (BC-2403) and AAV9 sh-Control (BC-0183) adenovirus were customized, synthesized, and purified by Shenzhen Brain Case Biotechnology Inc.

### 2.2 Animals

Ten-week-old male wild-type C57BL/6 J mice were purchased from GemPharmatech LLC and housed in a controlled environment at 22 ± 2°C with a 12-h light–dark cycle, receiving standard chow and water *ad libitum*. The mice were randomly assigned to receive a stereotactic injection of AAV9 sh-FSHR or AAV9 sh-Control. One week later, they were intraperitoneally injected with recombinant human FSH or normal saline (NS) once daily for a duration of 4 weeks. All of the mice are divided into six groups: FSH 0 IU group (normal saline 100 μL), FSH 5 IU group, FSH 10 IU group, sh-Control + NS group, sh-Control + FSH group (sh-Control +FSH 5 IU), and sh-FSHR + FSH group (sh-FSHR + FSH 5 IU), 12 mice per group. All animal procedures were conducted in accordance with the guidelines approved by the Institutional Animal Care and Use Committee (IACUC) of Renmin Hospital of Wuhan University, with approval number 20240501B.

### 2.3 Cells

The human neuroblastoma cell line SH-SY5Y was obtained from ATCC. SH-SY5Y was cultured in DMEM/F12 with 10% fetal bovine serum (v/v), penicillin (100 U/mL, w/v) and streptomycin (100 μg/mL, w/v). The cells were incubated at 37°C in a humidified atmosphere of 5% CO_2_. When the cells grow to the right density, the ERK inhibitor (10 nM) was added to culture medium, 30 min later, the cells were treated with FSH (30 ng/mL) for 48 h.

### 2.4 Stereotactic injection

Ten-week-old mice were anesthetized with isoflurane prior to receiving bilateral intracerebral injections of AAV9 sh-FSHR or AAV9 sh-Control. The stereotactic coordinates for injection were as follows: −1.5 mm anterior–posterior, −2.06 mm mediolateral from the bregma, and −1.85 mm dorsoventral. A volume of 200 nL of virus suspension containing 2 × 10^9^ vector genomes (vg) was bilaterally injected at a rate of 50 nL/min using a 10 μL glass syringe. The needle was left in place for 5 min post-injection before being slowly removed. Following the procedure, the mice were placed on a heated surface until they recovered from anesthesia.

### 2.5 Forced swimming test

The Forced Swimming Test (FST) was used to evaluate depressive behavior in mice by quantifying immobility duration. Each mouse was placed individually into a glass beaker with specific dimensions (inner diameter of 15 cm and depth of 35 cm) filled with warm water (25 ± 1°C) to a depth of 10 cm. The water was replaced after each test session. The mice were allowed to swim freely for a duration of 6 min, with the last 4 min being recorded to determine immobility time. Immobility was operationally defined as remaining motionless or floating with minimal movement required to maintain the head above water. An increase in immobility time was indicative of depression-like behavior when compared.

### 2.6 Tail suspension test

The tail suspension test (TST) was used to evaluate depressive behavior by measuring the duration of immobility for suspended mice. Each mouse was suspended 20 cm away from the floor, with adhesive tape placed 1 cm from the tail tip. Mice were considered immobility when they are passively suspended for 10 s without body movement. The behavior of the animals was recorded by a video camera during a 6 min period and more extended periods of inactivity indicated depressive behavior compared to control mice.

### 2.7 Sucrose preference test

The sucrose preference test (SPT) was used to evaluate anhedonia, which is one of the main characteristics of depression. Anhedonia was defined as a percentage of sucrose preference below 65%. A 6-day sucrose preference regimen was employed to detect depression-like behavior in mice after being raised individually for 1 week. Specifically, mice were provided with two bottles of regular water for 48 h and then were given 48 h of continuous exposure to two bottles, one containing sucrose water (2%) and one containing regular water for sucrose solution adaptation (switched the position of the two bottles halfway through). After being deprived of water for 24 h, the mice were again given two bottles, one containing sucrose water (2%) and one containing regular water and the consumption of each fluid for 12 h were recorded for analysis. The sucrose preference was expressed as a ratio of sucrose intake to total fluid intake.

### 2.8 Open field test

The open field test (OFT) was carried out in a plexiglass box (50 × 50 × 50 cm) to assess the anxiety activity. We divided the floor of the box into two parts: the central area of 35 × 35 cm and the surrounding area. On two consecutive days, mice were placed in a corner and allowed to explore the chamber freely for 5 min. The camera was placed 180 cm above the device center for tracking the mouse’s motion, and ANY Maze software recorded the data. The system measured the duration of time the mouse spent in the central area and the frequency of entries into the central location. The parameters of the second-day test were employed to evaluate behaviors associated with anxiety. The decrease in both central time and central entry frequency suggested anxiety-related behaviors in comparison to the control group of mice.

### 2.9 Elevated plus maze

The elevated plus-maze (EPM) test was used to evaluate anxiety-like behavior. The elevated plus-maze consists of a plus-shaped platform, with two open arms (30 × 5 × 0.5 cm) and two closed arms (30 × 5 × 15 cm), which is 40 cm above the floor. At the beginning of the test, the mice were placed in the central area facing the open arm. During the five-minute experiment, the mice’s movements were tracked by a camera above the maze’s center and recorded with ANY-maze software. It was calculated as the percentages of open arm entries divided by the total number of arm entries and the time spent in the open arms divided by the total time, respectively.

### 2.10 Novel object recognition

The novel object recognition test (NOR) was performed in an open-field apparatus (50 × 50 × 50 cm) for evaluating the hippocampal-dependent memory. The mice were given 10 min to freely explore the equipment before the test. On the initial day, the mice were exposed to two identical objects (familiar objects) that were situated in the left and right corners of the space, and the mice were permitted to freely explore the object for 5 min. One of the t objects was replaced by a novel object on the second day and the mice were permitted to explore for 5 min without any restriction. The mice’s exploration of both familiar and new objects was captured by a digital camera and the parameters were recorded with the ANY-maze software. “Exploring” was defined as touching the object (with the exception of its tail) or sniffing it (with a distance of less than 2 cm). We used the discrimination index to examine recognition ability by dividing the amount of time spent exploring the novel object by the amount of time spent exploring familiar and novel objects. The intact spatial recognition memory can be indicated by a preference for the novel object.

### 2.11 Immunostaining

We used 4 μm paraffin-embedded brain sections for immunofluorescence staining. The sections were deparaffinized and hydrated. And then, antigen retrieval buffer (0.1 M sodium citrate, pH 6.0) was applied to sections for 20 min at 94°C, followed by a 10 min treatment with 3% hydrogen peroxide to remove nonspecific background staining, and the sections were washed 3 times in PBS. Then, the sections were incubated overnight at 4°C with primary anti-GFAP (1:300), anti-Iba1 (1:300), anti-FSHR (1:200), anti-GluR1 (1:200), and anti-GAD67 (1:200) antibodies. After washing with TBS for 3 times, the sections were incubated with secondary antibodies at room temperature in darkness for 2 h for detection. After a 3-time wash with TBS, DAPI was used for staining nuclei. The images were acquired by the Leica confocal imaging system.

### 2.12 Western blotting

The hippocampus of mice was lysed in cold RIPA lysis buffer containing protease and phosphatase inhibitors, followed by an incubation of 30 min on ice. Then, we centrifuged the sample for 25 min at 15,000 rpm, and then collected the supernatant. The protein concentration was quantified with BCA Protein Assay kit (Thermo Fisher Science, A55860). The samples were transferred to nitrocellulose membranes after SDS-PAGE. Blocked the membranes in blocking buffer containing 5% nonfat milk and 0.1% Tween-20 Tris-buffered saline (TBS-T) at room temperature for 2 h, followed by incubation overnight at 4°C with primary antibodies against IL-1*β* (1:1,000), IL-6 (1:500), PSD95 (1:1,000), synapsin (1:1,000), synaptophysin (1:1,000), FSHR (1:1,000), GluR1 (1:2,000), GluR2 (1:1,000), VGluT1 (1:1,000), GAD67 (1:1,000), β-actin (1:2,000), ERK (1:1,000), p-ERK (1:1,000). After being washed with TBS-T for 5 times, the membrane was incubated for 1 h at room temperature with secondary antibody. The membranes were developed in enhanced chemiluminescent (ECL) detection system, and images were collected by the Bio-Rad gel imaging system.

### 2.13 ELISA

Mouse blood was collected and centrifuged (2,500 rpm, at 4°C for 5 min), and the serum was collected and stored at −80°C. According to the manufacturer’s instructions, FSH, LH, 17β-estradiol, IL-1β, IL-6, and TNF-*α* in mouse serum were measured by specific human FSH ELISA kit, mouse LH ELISA kit, mouse 17β-estradiol ELISA kit, mouse IL-1β ELISA kit, mouse IL-6 ELISA kit, and mouse TNF-α ELISA kit.

### 2.14 Transmission electron microscopy

Under isoflurane anesthesia, the mouse hippocampus was rapidly isolated after cardiac perfusion with electron microscope fixation (Servicebio, G1102-100ML). Mouse hippocampus was chopped into 1 × 1 × 1 mm cube with a sharp blade and then quickly placed them into test tubes filled with electron microscope fixative. The samples were stored in a dark place at room temperature for 2 h and then at 4°C. Our method involved staining the ultrathin sections (90 nm) with uranyl acetate and lead acetate and then observing them using a JEOL 200CX Electron Microscope at 100 kV. Synapses were detected by observing their presence of synaptic vesicles and postsynaptic densities. Synapse density in area CA1 of the hippocampus was calculated.

### 2.15 Golgi staining

Mice brains were subjected to a 24-h fixation with 10% formalin, followed by immersion in 3% potassium bichromate for 3 days in darkness, and we changed the potassium bichromate solution daily. Thereafter, the brains were moved to a solution containing 2% silver nitrate and kept in darkness for 7 days. We then cut the brain tissue into slices of 30 μm using a vibratome and air dried them for 10 min. Subsequently, the slices were dehydrated with 95 and 100% ethanol, cleaned with xylene, and finally sealed with coverslips. Bright-field images of pyramidal neurons in the hippocampus and cortex were taken at 100X magnification using a Zeiss Axioplan (Zeiss, Decatur, GA, United States) microscope. The number of spines were calculated using ImageJ software.

### 2.16 LC–MS/MS analysis

After 1 week of continuous intraperitoneal injection of FSH (*n* = 3 mice) or NS (*n* = 3 mice), mouse brain samples were taken for proteomic detection. The peptides were dissolved in mobile phase A by liquid chromatography and separated by EASY-nLC 1,200 ultra-high-performance liquid phase system, and then the nano spray ionization ion source was injected into them for ionization, finally they were analyzed by Orbitrap Exploris 480 mass spectrometry. MaxQuant with the Andromeda search engine (v1.4.1.2) integrated was selected for database searching of the resulting MS/MS data. After the database search was completed, the quality control evaluation, quantitative analysis and differentially expressed proteins (DEPs) screening of the mass spectrum results were carried out.

### 2.17 Bioinformatic analysis

Kyoto encyclopedia of genes and genomes (KEGG) pathway enrichment analysis was employed to test the enrichment of the DEPs against the identified protein, and the top 20 items analyzed by KEGG were presented as histograms. The results were visualized using the “cluster profiler” packages of R software and the OmicShare tools.^1^

### 2.18 Statistical analysis

Statistical analyses and data diagram creation were performed using GraphPad Prism 8.3 software. The tests were analyzed by either unpaired two-tailed Student’s *t*-test for two-group comparison or one-way ANOVA (followed by Tukey’s or Brown-Forsythe and Welch) test for multiple groups comparison. Differences with *p* < 0.05 were considered statistically significant.

## 3 Results

### 3.1 FSH enhanced anxiety and depression-like behavior, as well as memory impairment in mice

**Figure 1:**
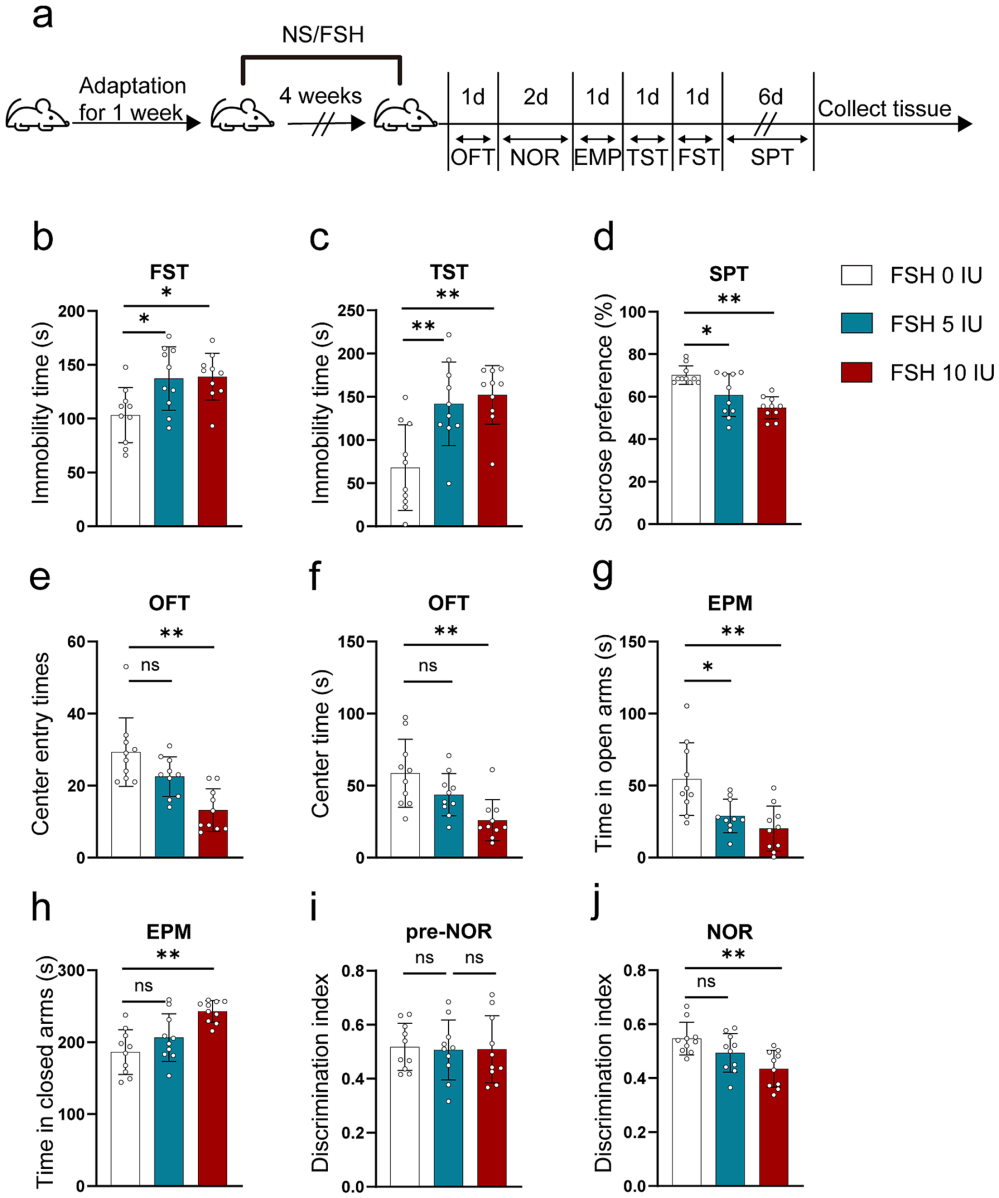
FSH induces depressive behaviors in mouse. 10-week-old C57BL/6 J male mice were randomly treated with different dose of recombinant human FSH (0 IU, 5 IU, or 10 IU per day, 6 days a week) continuously by intraperitoneal injection for 4 weeks. (a) Schematic diagram of the animal experiment. (b) Immobility duration in the forced swim test (FST). (c) Immobility duration in the tail suspension test (TST). (d) Sucrose preference in the sucrose preference test (SPT). (e,f) The center entry times and center time in the open-field test (OFT). (g,h) The time spent in the closed arms and open arms in the elevated plus maze (EPM) test. (i,j) The discrimination index in the novelty object recognition (NOR) test. Data are presented as the mean ± SEM, *n* = 10 mice for each group, one-way ANOVA followed by Tukey’s multiple comparisons test. *p < 0.05, **p < 0.01; ns, not significant.

To explore whether FSH can induce behavioral alterations in mice, we gave C57BL male mice different doses of recombinant human FSH protein by intraperitoneal injection (0 IU/5 IU/10 IU, once a day) for 4 weeks. Then we evaluated anxiety and depressive behaviors via FST, TST, SPT, OFT, and EPM. Figure 1a shows the schematic diagram of the animal experiment. The results reflected that FSH dose-dependently induced extended immobility time in FST and TST (Figures 1b,c). Meanwhile, the result from SPT showed that mice showed a lower sucrose preference than control mice after being treated with 5 IU or 10 IU FSH, suggesting that FSH induced depression-like behavior in mice (Figure 1d). In OFT, we found that the number of center entries and center entry time decreased after FSH (10 IU) treatment in mice (Figures 1e,f), and the results from EPM test showed that the mice spent considerably less time in the open arms and significantly more time in the close arms after FSH treatment (Figures 1g,h), which represented FSH dose-dependently induced anxiety-like behavior. NOR was used to assess visual recognition memory. The findings from NOR test revealed that the mice were less active in exploring new objects after FSH (10 IU) treatment (Figures 1i,j). Taken together, these findings suggest that mice exposed to FSH had increased levels of anxiety and depression and impaired memory.

Of notes, to assess the impact of FSH injection on hormone fluctuation in mice, the serum levels of humanized FSH, LH, and 17β-estradiol were measured in female mice. The results indicated that injections with 5 IU or 10 IU FSH significantly increased humanized FSH levels (Supplementary Figure S1a) but had little effect on LH and 17β-estradiol levels (Supplementary Figures S1b,c), suggesting that FSH-induced depressive behavior in mice was independent on estrogen deficiency.

### 3.2 FSH induced inflammation, synaptic plasticity impairment, and glutamate/GABA cycle disturbance in mice

**Figure 2:**
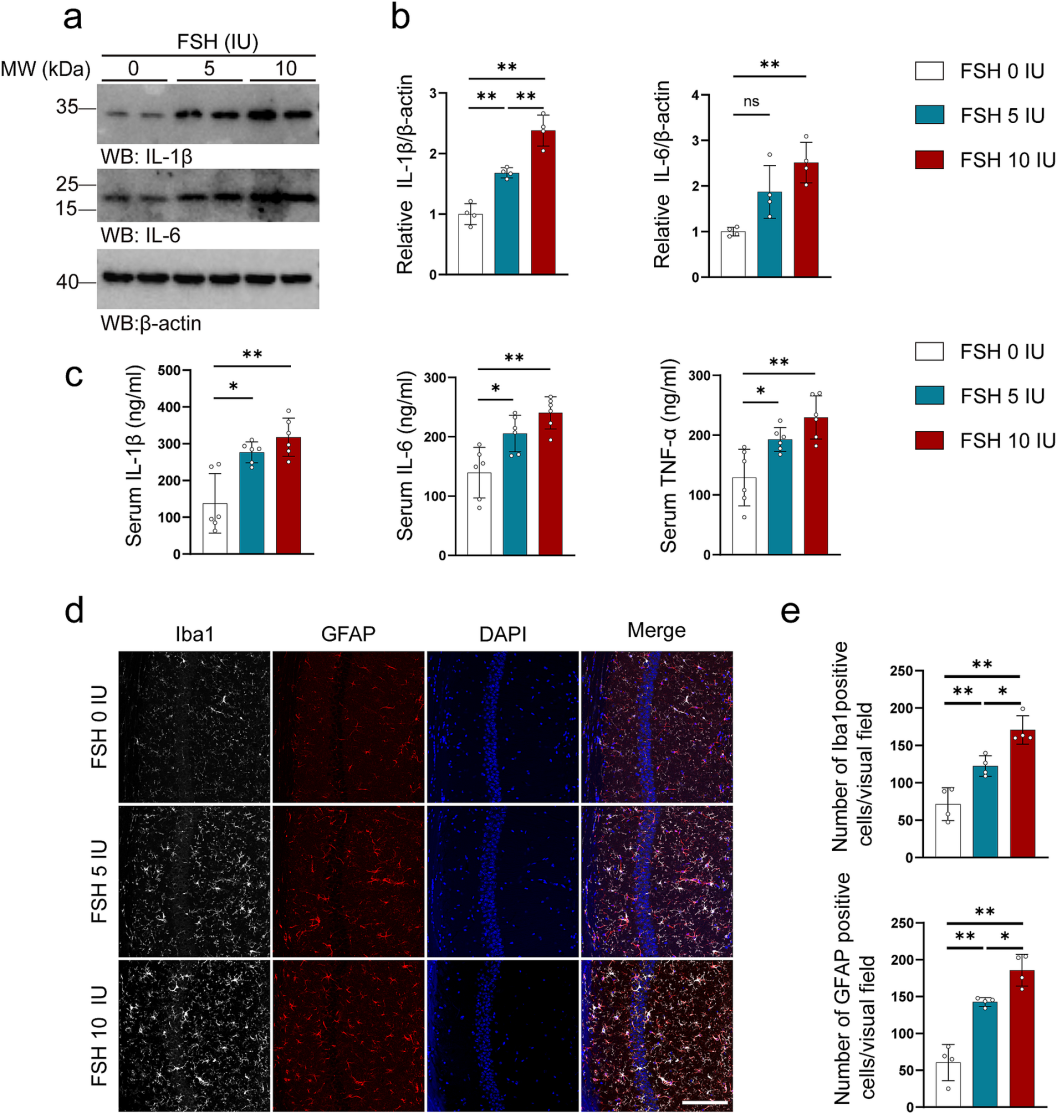
FSH induces neuroinflammation in mice. Four weeks after the FSH treatment, the brain and serum of the mice were harvested for western blot (a,b), Elisa assay (c), and immunofluorescence staining (d,e). (a) Representative immunoblots showing the protein expression of IL-1β and IL-6 in the hippocampus. (b) Quantification of the immunoreactivity of IL-1β and IL-6 protein expression, data are shown as represent mean ± SEM, *n* = 4 mice per group. (c) ELISA quantification shows the levels of proinflammation factors IL-1β, IL-6, and TNFα in the serum, data are shown as mean ± SEM, *n* = 6 mice per group. (d) Immunofluorescent co-staining of Iba1 (gray) and GFAP (red) on the hippocampal sections (scale bar: 150 μm). (e) Quantification of Iba1 positive and GFAP positive cells, data represent mean ± SEM (*n* = 4 mice per group, 10 sections each mouse). Statistical analysis was performed by one-way ANOVA followed by Tukey’s multiple comparisons test. *p < 0.05, **p < 0.01; ns, not significant.

In addition to behavioral tests, we also examined the pathological changes associated with depression in mice. It has been documented that neuroinflammation plays a vital role in the etiology of mood disorders (Troubat et al., 2021). We first tested the level of inflammation in untreated mice and mice treated with intraperitoneal injection of NS and found that intraperitoneal injection of NS had no effect on the expression of IL-1β in the hippocampus of mice compared with the untreated group (Supplementary Figures S2a,b). As shown in Figure 2, we explored the neuroinflammatory alternations in mice after FSH administration. First, the proinflammatory cytokines (IL-1β, IL-6) expression were significantly increased after FSH treatment in a dose-dependent manner (Figures 2a,b). Second, the serum levels of IL-1β, IL-6, and TNF-*α* also exhibited an increase after FSH treatment (Figure 2c), irrespective of the dosage administered (5 IU or 10 IU). At last, the astrocyte and microglia activation state in hippocampus were detected by GFAP and IBA1 immunofluorescence staining, and we found that the number of Iba1 and GFAP-positive cells in the hippocampus increased in correlation with the dosage of FSH administered (Figures 2d,e). These findings suggest that FSH causes heightened levels of neuroinflammation in mice.

**Figure 3:**
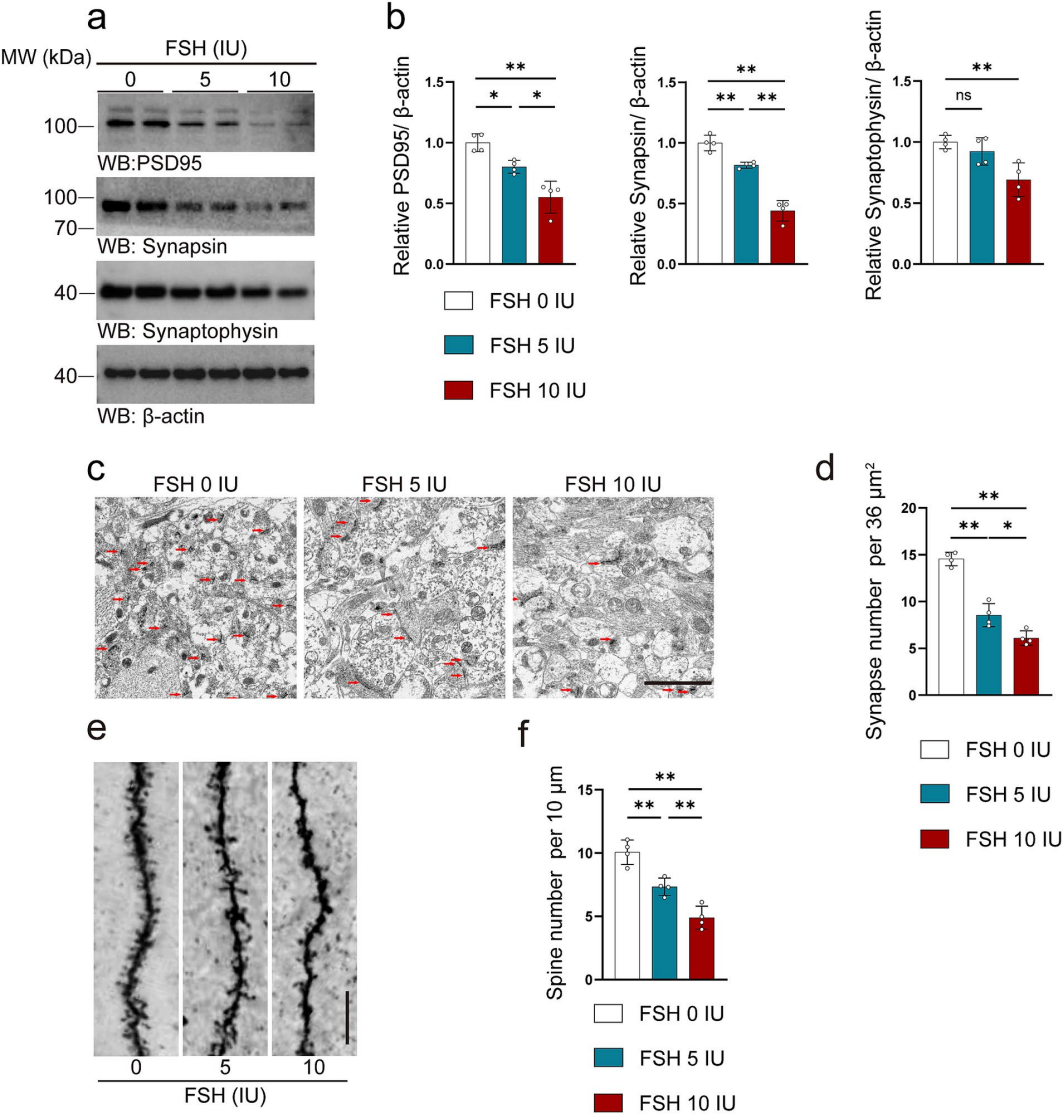
FSH induces synaptic plasticity impairment in mice. (a) Representative images of western blot showing PSD95, synapsin and synaptophysin expression after FSH treatment. (b) Quantification of protein expression, *n* = 4 mice per group. (c,d) Transmission electron micrographs images (c) and quantification (d) of synapses in hippocampal sections after FSH treatment, the red arrows represent synapses (scale bar, 2 μm), statistic data are shown as mean ± SEM (*n* = 4 mice per group, 8 different visual fields each mouse). (e) Golgi staining revealed fewer density of dendritic spines from the apical dendritic layer of the CA1 region after FSH treatment (scale bar, 10 μm). (f) Quantification of the dendritic spine density, which is calculated as the number of dendritic branches per 10 μm dendrite, data are presented as mean ± SEM, *n* = 4 mice per group. Statistical analysis was performed by one-way ANOVA followed by Tukey’s multiple comparisons test. *p < 0.05, **p < 0.01; ns, not significant.

The synaptic structure serves as the foundation for neuronal function, with synaptic plasticity impairment being strongly associated with depressive behaviors (Duman et al., 2016). As shown in Figure 3, FSH induced synaptic plasticity impairment in hippocampus of the mice. We evaluated the crucial markers of synapses via western blotting, the data showed the expression levels of PSD95, synapsin, and synaptophysin were decreased with the increase of FSH dose (Figures 3a,b). Moreover, transmission electron microscopy of synapses in hippocampus showed that the synapses number were declined after FSH treatment, especially in the FSH 10 IU group (Figures 3c,d). Consistent with this, Golgi staining showed that FSH treatment impaired the dendritic spines in the hippocamps (Figures 3e,f). The implication of these results is that high dose of FSH stimulation triggers synaptic plasticity impairment.

**Figure 4:**
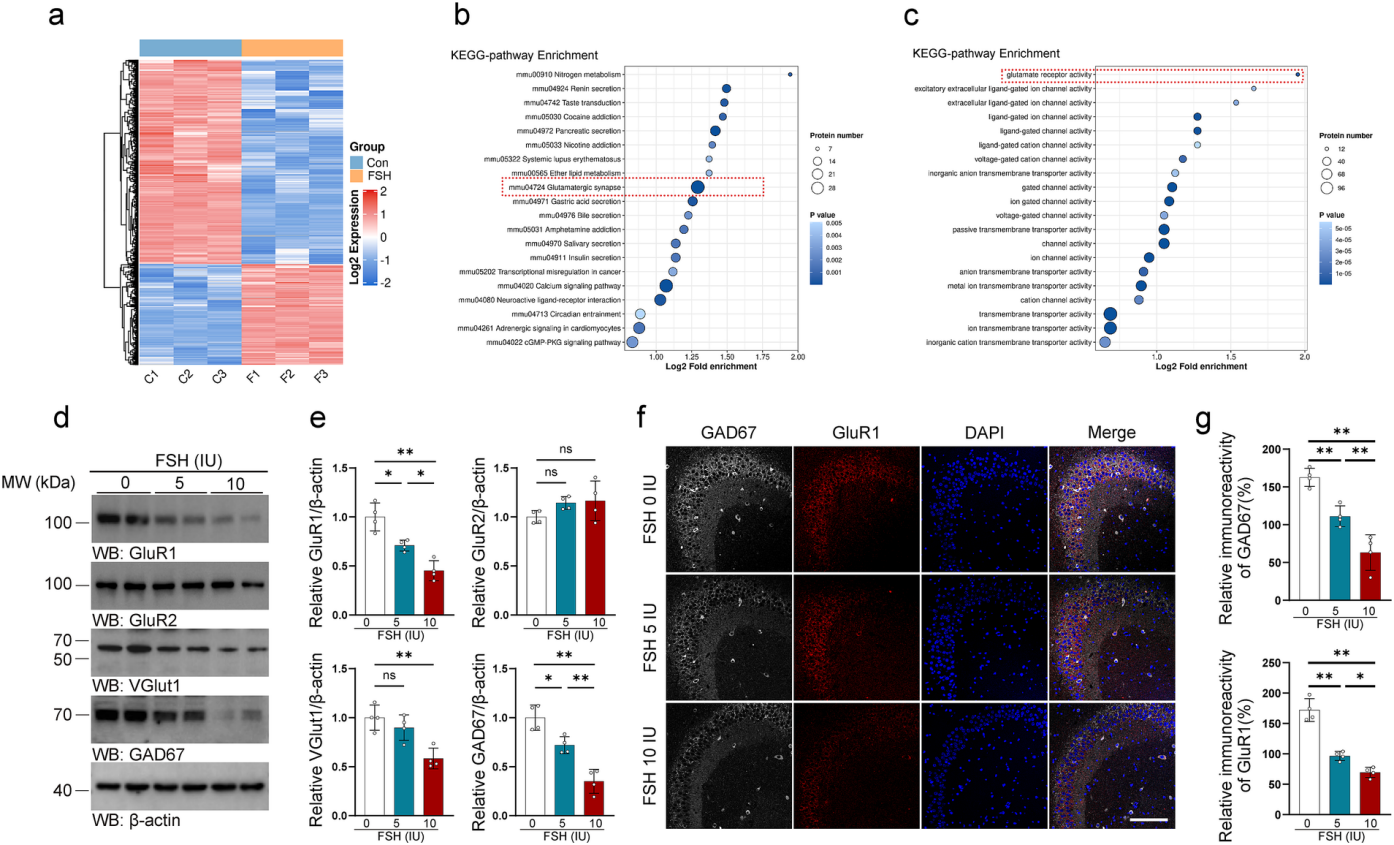
FSH induces glutamate/GABA cycle disturbance in mice. (a–c) Proteomics detection of different protein expression in the brain tissue after continuously FSH treatment for 7 days. Heat map of differential expression of FSH-induced protein (a), Bubble plot of KEEG enrichment analysis reveals significant differences in the protein expression associated to glutamatergic synapse (b) and glutamate receptor activity (c), *n* = 3 mice per group. (d) Representative immunoblots images showing the protein expression of GluR1, GluR2, VGlut1, GAD67 after FSH treatment for 4 weeks. (e) Quantification of the immunoreactivity in (d), data are presented as mean ± SEM, *n* = 4 mice per group. (f) Immunofluorescent co-staining of GAD67 (gray) and GluR1 (red) on the hippocampal sections (scale bar, 150 μm). (g) The immunoreactivities of GAD67 and GluR1 were quantified, data are presented as mean ± SEM (*n* = 4 mice per group, 10 sections each mouse). Statistical analysis was performed by one-way ANOVA followed by Tukey’s multiple comparisons test for (d)/GluR1, VGlut1, GAD67 quantification and (g) quantification, Brown-Forsythe and Welch test for (d)/GluR2 quantification. *p < 0.05, **p < 0.01; ns, not significant.

Disturbances in neurotransmitter activity with the brain are known to associate with depression (Lener et al., 2017; Fries et al., 2023). More precisely, abnormal signaling of glutamate and gamma-aminobutyric acid (GABA) are thought to play a role in depression (Garcia-Garcia et al., 2009; Luscher et al., 2023; Duman et al., 2019). Therefore, we tested glutamatergic and GABAergic system-related indicators in mice treated with FSH. We first conducted proteomics tests on brains of the mice following intraperitoneal injection of FSH for 1 week, and then performed KEGG analysis to analyze the protein alternation induced by FSH administration. The results revealed significant differences in the protein expression associated to glutamatergic synapse and glutamate receptor activity in the brains of the mice (Figures 4a–c). Based on this, we conducted an experiment to investigate the impact of FSH administration on the expression of GluR1, GluR2, VGluT1, and GAD67 proteins in the hippocampus of mice by western blotting. The results demonstrated that the protein levels of GluR1, VGluT1, and GAD67 in the hippocampus of mice were shapely declined after FSH treatment in a dose-dependent manner, and GluR2 protein expression showed no significant change (Figures 4d,e). Concurrently, immunofluorescence staining revealed a reduction in GluR1 and GAD67 fluorescence intensity in the FSH 5 IU and 10 IU groups as compared to the control group (Figures 4f,g). Given the above evidence, FSH treatment in mice resulted in neuroinflammation, hippocampal synaptic plasticity impairment, and glutamate/GABA cycle disorders.

### 3.3 FSHR knockdown alleviates levels of anxiety and depression and impaired memory induced by FSH

**Figure 5:**
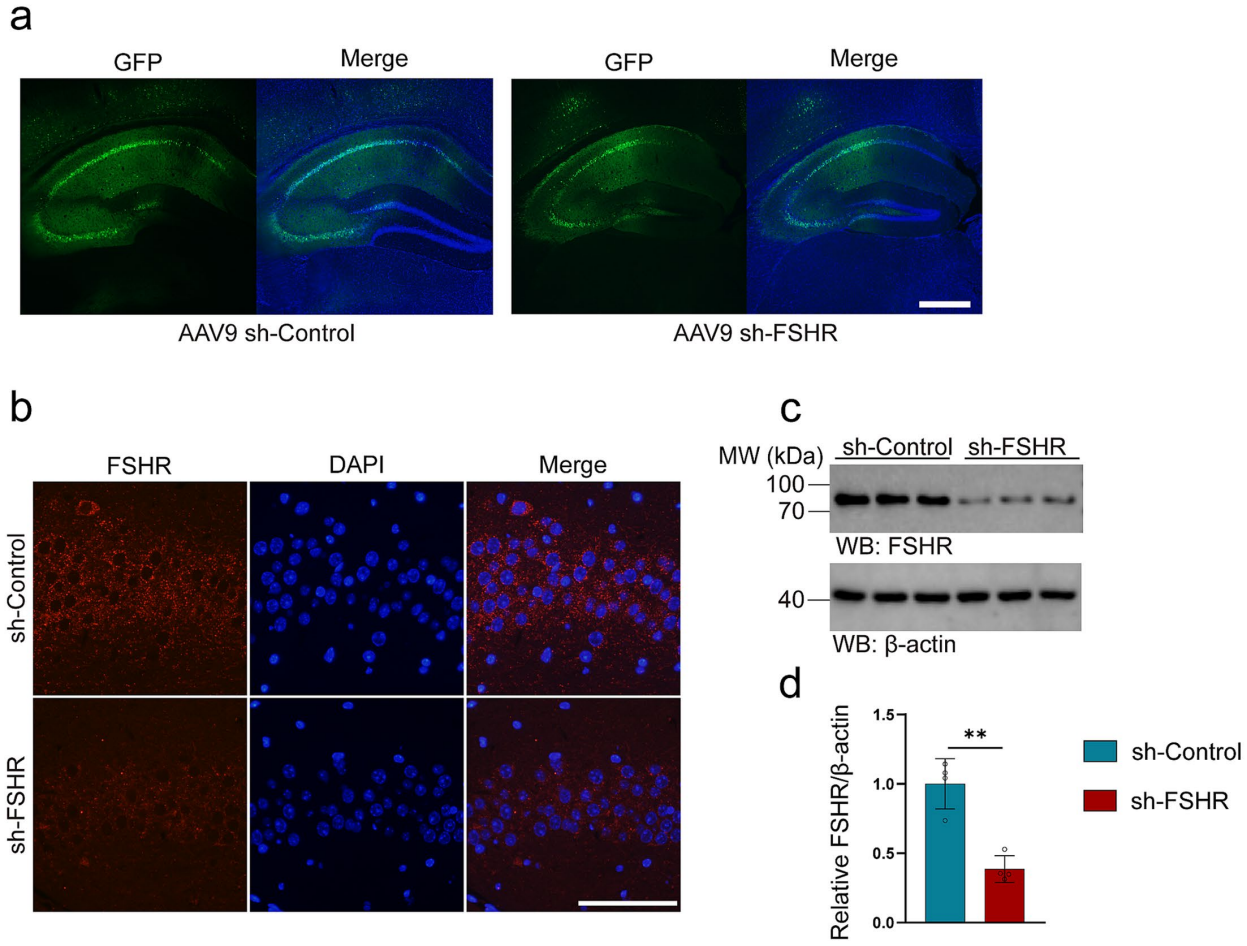
AAV9-adenovirus expression in the hippocampus of mice. In order to conditionally alleviated the effects of FSH in hippocampus of the mice, we knock-down the expression of FSHR in neurons of hippocampus by stereotactic injection of AAV9 sh-FSHR versus AAV9 sh-Control. The mouse brain was sampled 2 weeks later to verify the expression of adenovirus. (a) The expression of AAV9 sh-Control and AAV9 sh-FSHR adenovirus in mouse hippocampus (scale bar: 200 μm). (b) Immunofluorescent staining of FSHR (red) shows a reduced fluorescence intensity of FSHR on the hippocampal sections of the mice after AAV9-sh-FSHR injection (scale bar: 100 μm). (c,d) Representative immunoblots (c) and quantification (d) of FSHR protein expression in the hippocampus from the above mice, data are presented as mean ± SEM, *n* = 4 mice for each group. Statistical analysis was performed by unpaired two-tailed Student’s *t*-test, **p < 0.01.

The hippocampus is a key area of the brain associated with emotional regulation (Xiong et al., 2022; Bi et al., 2020a). It has been reported that patients with depression exhibit neurological dysfunction in the hippocampus (Gu et al., 2018a). Significantly, the hippocampus of mice has been found to express the FSHR (Xiong et al., 2022; Bi et al., 2020a). To investigate whether FSH induces the behavioral alterations and pathological changes by binding to FSHR in the hippocampus, we partially blocked FSH function by conditionally knockdown the FSHR expression in the neurons of the mouse hippocampus by stereotaxic injection of AAV9 sh-FSHR. Subsequently, mice were administered FSH daily for 4 weeks, which starts on 1 week after stereotactic injection. The mice were randomly divided into three groups: sh-Control + NS group, sh-Control + FSH group, sh-FSHR + FSH group. First, we ensure the accuracy of the injection site and the infection efficiency of AAV9 adenovirus by immunostaining (Figure 5a). Meanwhile, we verified a reduction in FSHR expression in mice injected with sh-FSHR in the hippocampus through immunostaining (Figure 5b) and western blotting (Figures 5c,d).

**Figure 6:**
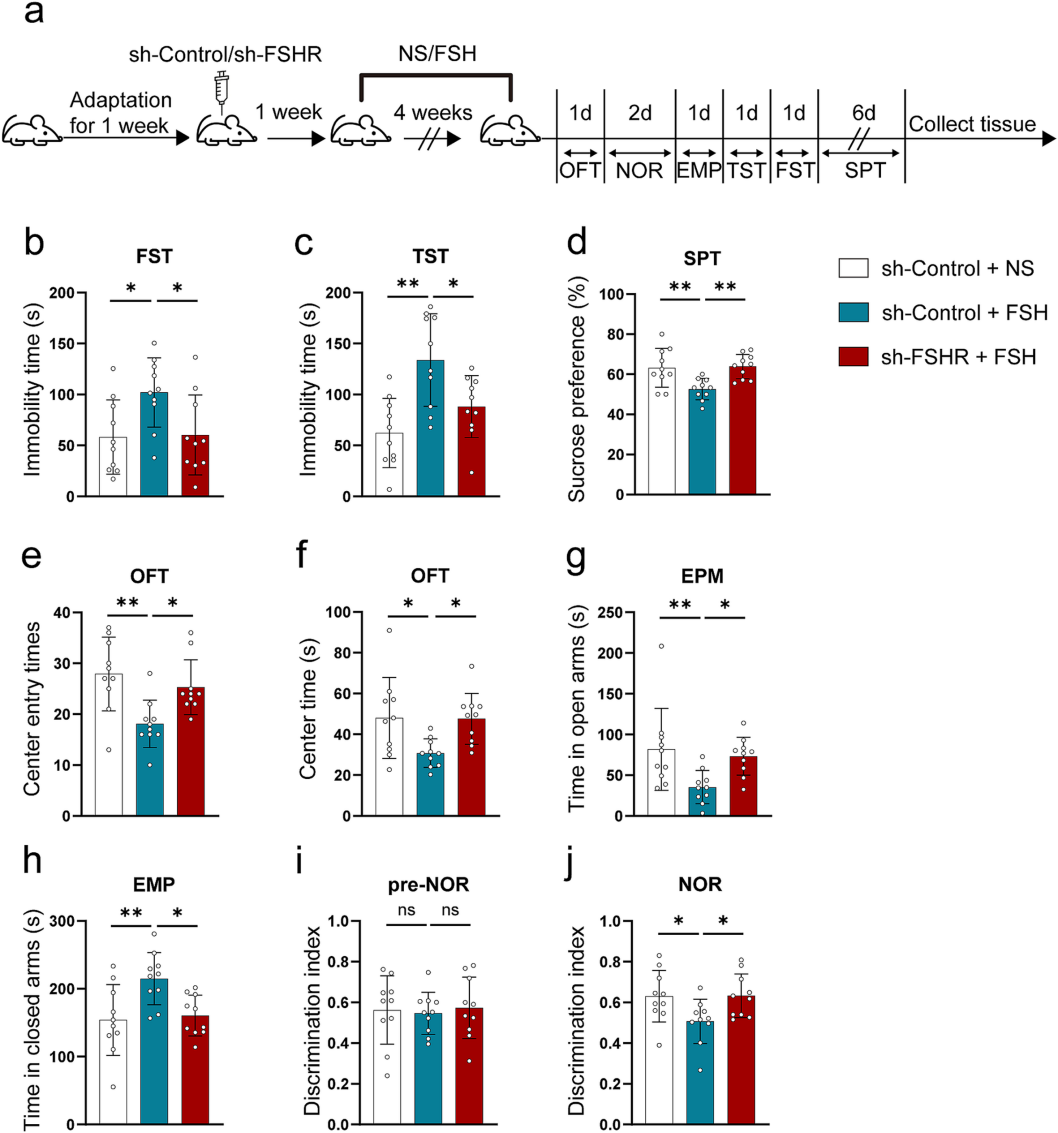
Knockdown of FSHR alleviates FSH-induced depressive behaviors in mice. AAV9 sh-Control or AAV9 sh-FSHR were injected into the hippocampus of 10-week-old C57BL/6 J male mice by stereotaxic injection. One week later, FSH or normal sodium (NS) were injected to the mice by intraperitoneal injection for 4 weeks. Mice were randomly divided into three groups: sh-Control + NS group, sh-Control + FSH group and sh-FSHR + FSH group. (a) Schematic diagram of the animal experiment. (b) Immobility duration in the FST. (c) Immobility duration in the TST. (d) Sucrose preference in the SPT. (e,f) The center entry times and center time in the OFT. (g,h) The time spent in the closed arms and the open arms in the EPM test. (i,j) The discrimination index in the NOR test. Data are presented as the mean ± SEM, *n* = 10 mice for each group. Statistical analysis was performed by one-way ANOVA followed by Tukey’s multiple comparisons test. *p < 0.05, **p < 0.01; ns, not significant.

We performed behavioral tests on mice. Figure 6a shows schematic diagram of the animal experiment. The results of FST and TST revealed that knockdown of FSHR resulted in a decrease in immobility time when compared to the control group without FSHR knockdown (Figures 6b,c). Additionally, the results in SPT demonstrated a higher preference for sucrose in mice of sh-FSHR + FSH group compared to those in sh-Control + FSH group (Figure 6d). Furthermore, the results from OFT indicated that mice in sh-FSHR + FSH group exhibited increased center entries (Figure 6e) and spent more time in the center (Figure 6f) compared with sh-Control + FSH group. In EPM test, mice in sh-FSHR + FSH group spent significantly more time in the open-arms and substantially less time in the closed-arms compared to sh-Control + FSH group (Figures 6g,h). NOR were performed to evaluate the memory impairment in the mice with or without FSHR knockdown. We found that mice in sh-FSHR + FSH group showed a longer period of time to explore novel things compared with sh-Control + FSH group, suggesting that FSHR knockdown mitigated FSH-induced memory impairment in mice (Figures 6i,j). In summary, our findings revealed that FSHR knockdown alleviates FSH-induced anxiety and depressive behaviors and memory impairment in mice.

### 3.4 FSHR knockdown alleviated FSH-induced neuroinflammation, synaptic plasticity impairment, and glutamate/GABA cycle disturbance in mice

**Figure 7:**
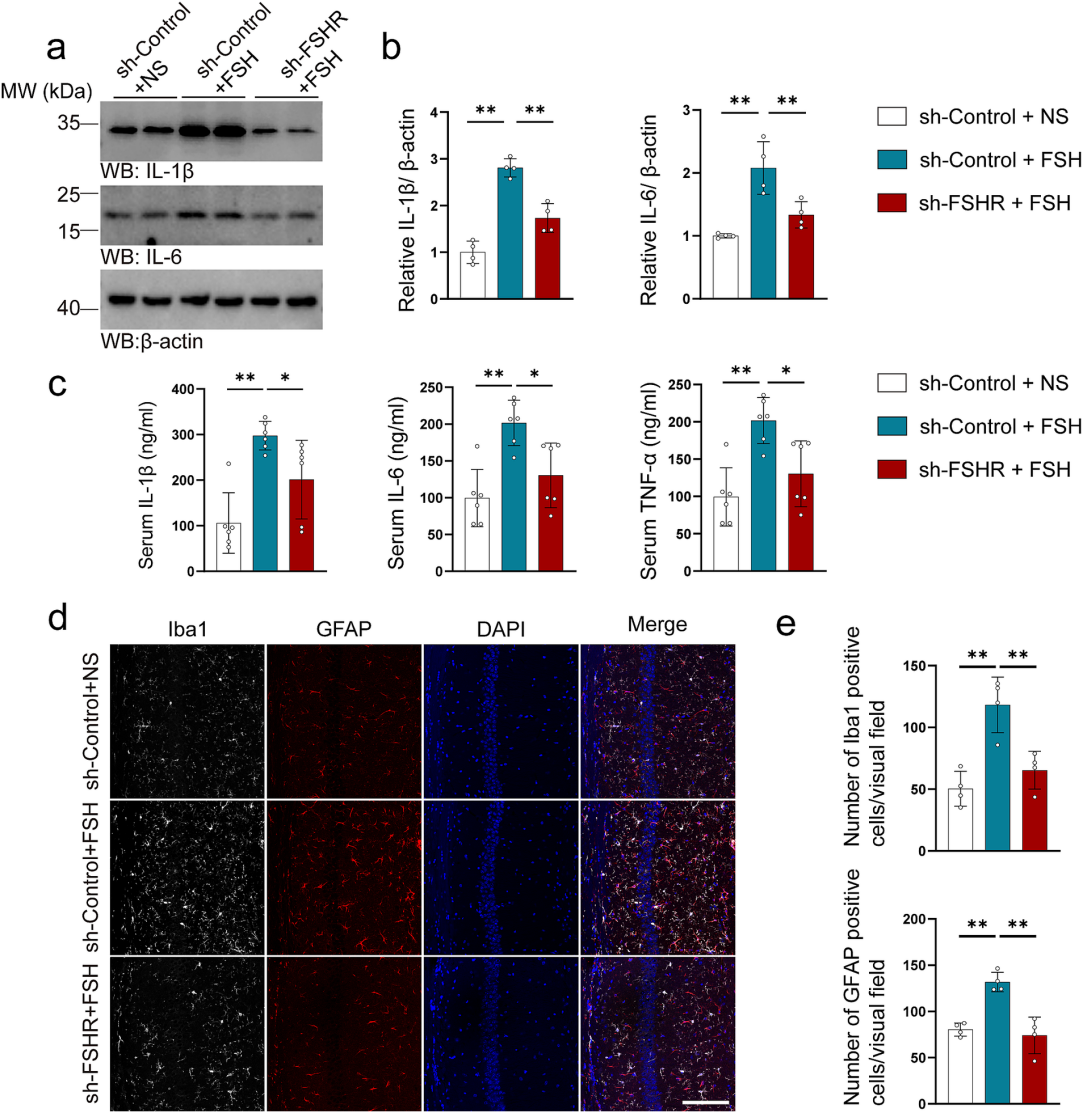
FSHR knockdown alleviates FSH-induced neuroinflammation in mice. (a,b) Representative immunoblots images (a) and quantification (b) of IL-1β and IL-6 protein expression showing a reduction in the hippocampus of the sh-FSHR + FSH group compared to sh-Control + FSH group, data are presented as mean ± SEM, *n* = 4 mice per group. (c) ELISA quantification of pro-inflammation factors IL-1β, IL-6, and TNFα in the serum, data are presented as mean ± SEM, *n* = 6 mice per group. (d,e) Immunofluorescent co-staining (d) and quantification (e) of Iba1 (gray) and GFAP (red) on the hippocampal sections (scale bar: 150 μm), data are presented as mean ± SEM (*n* = 4 mice per group, 10 sections each mouse). Statistical analysis was performed by one-way ANOVA followed by Tukey’s multiple comparisons test, *p < 0.05, **p < 0.01.

**Figure 8:**
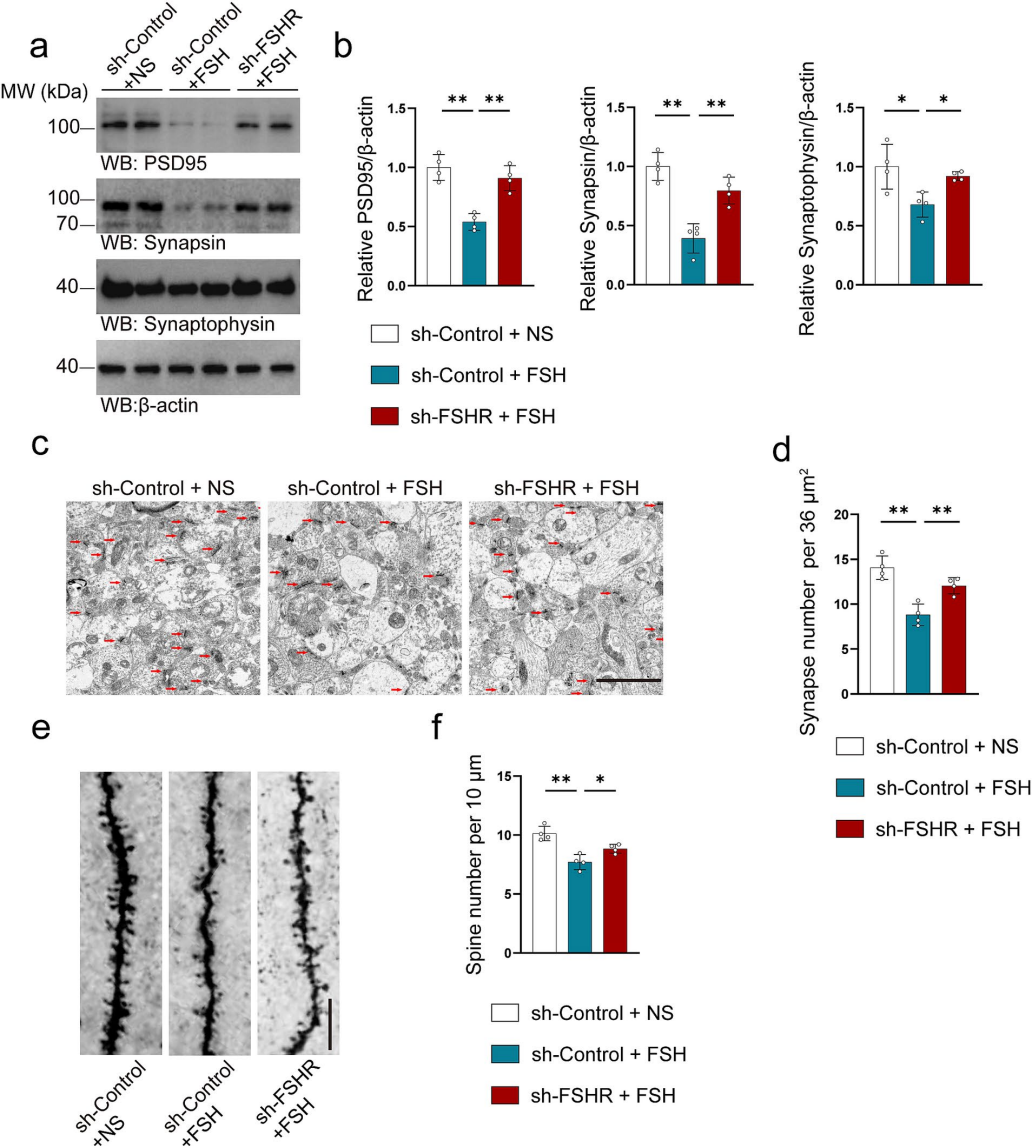
FSHR knockdown alleviates FSH-induced synaptic plasticity impairment. (a,b) Representative immunoblots images (a) and quantification (b) of the protein expression of PSD95, synapsin, and synaptophysin in hippocampus, data are presented as mean ± SEM, *n* = 4 mice per group. (c,d) Transmission electron micrographs images (c) and quantification (d) of synapse in hippocampal sections showing better synapse morphology in the sh-FSHR+FSH group than in the sh-Control + FSH group (scale bar, 2 μm), data are presented as mean ± SEM (*n* = 4 mice per group, 8 views each mouse). (e,f) Golgi staining (e) and quantification (f) of dendritic spines showing a less reduction of dendritic spine from the apical dendritic layer of the hippocampus in the sh-FSHR + FSH group than in the sh-Control + FSH group (scale bar, 10 μm), data are shown as mean ± SEM, *n* = 4 mice per group. Statistical analysis was performed by one-way ANOVA followed by Tukey’s multiple comparisons test, *p < 0.05, **p < 0.01.

We further explored the effect of FSHR knockdown in hippocampus on FSH-induced pathological changes by western blotting, immunofluorescence, ELISA, synaptic electron microscope, and Golgi staining. Regarding FSH-induced neuroinflammation, the results showed that FSHR knockdown attenuated the FSH-induced upregulation of IL-1β and IL-6 expression levels (Figures 7a,b). The results of ELISA indicated that the elevated serum levels of proinflammatory cytokines (IL-1β, IL-6, TNF-*α*) induced by FSH were significantly reduced following FSHR knockdown (Figure 7c). And moreover, the number of Iba1 and GFAP-positive cells in hippocampus of sh-FSHR + FSH group were less than that in sh-Control + FSH group in immunofluorescence (Figures 7d,e). In terms of synaptic plasticity impairment, western blotting analysis showed that the expression levels of PSD95, synapsin, and synaptophysin in sh-FSHR + FSH group were higher than those in sh-Control + FSH group (Figures 8a,b); moreover, transmission electron microscopy of synapses in hippocampus showed that the synapses number in the sh-FSHR + FSH group were more than that in the sh-Control + FSH group (Figures 8c,d); and Golgi staining showed that FSHR knockout could partially protect the hippocampal neurons from FSH-induced loss of dendritic spines (Figures 8e,f).

**Figure 9:**
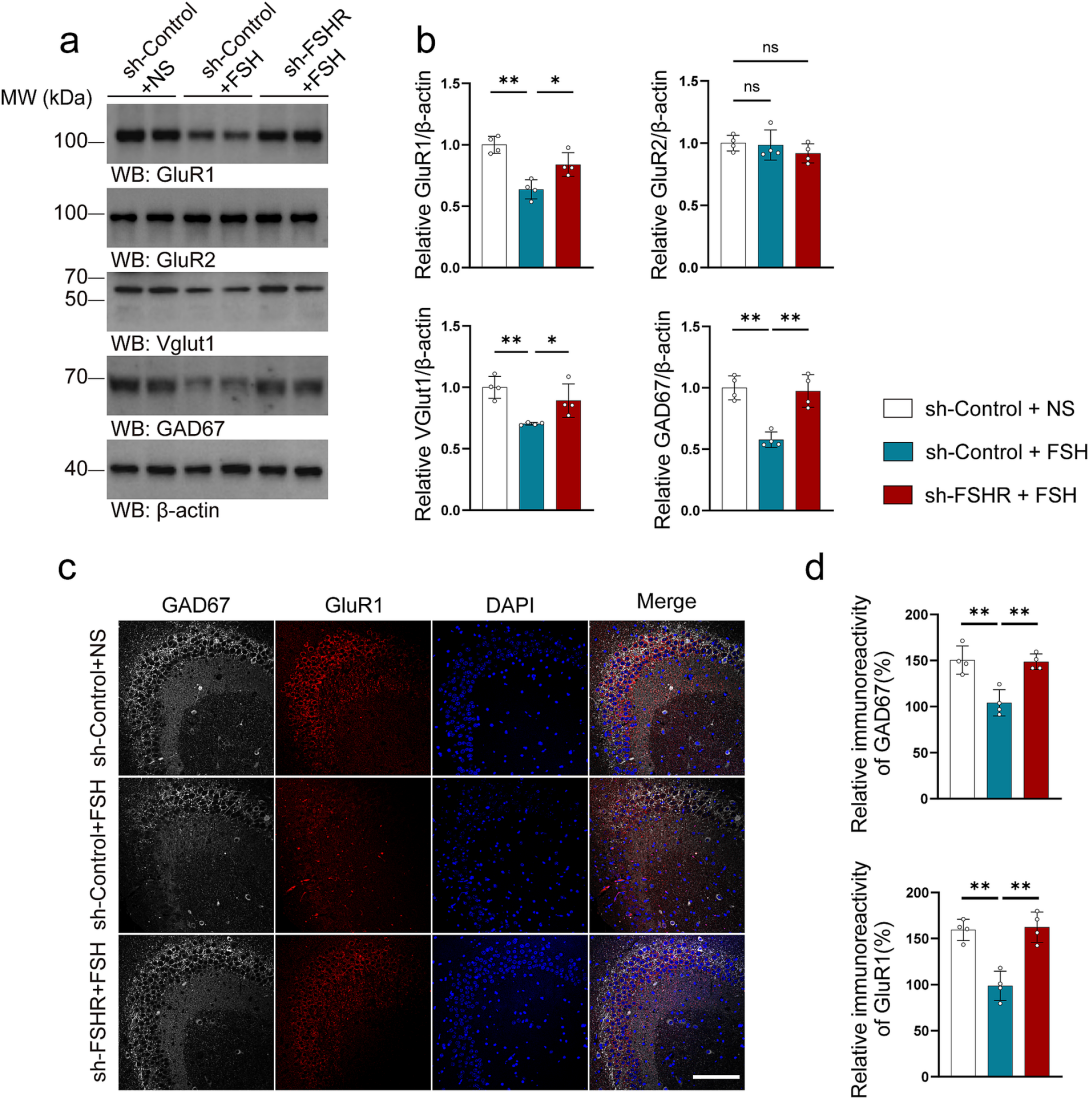
FSHR knockdown alleviates FSH-induced glutamate/GABA cycle imbalance in mouse brain. (a,b) Representative immunoblots images (a) and quantification (b) of GluR1, GluR2, VGlut1, GAD67 protein expression in the hippocampus, data are presented as mean ± SEM, *n* = 4 mice per group. (c,d) Immunofluorescent co-staining (c) and quantification (d) of GAD67(gray) and GluR1(red) on the hippocampal sections (scale bar, 150 μm), data are presented as mean ± SEM (*n* = 4 mice per group, 10 sections each mouse). Statistical analysis was performed by one-way ANOVA followed by Tukey’s multiple comparisons test. *p < 0.05, **p < 0.01; ns, not significant.

In addition, we tested the related indexes of glutamate and GABA neurotransmitter system in mouse hippocampal neurons. We found that the lowered expression levels of GluR1, VGluT1, and GAD67 induced by FSH were alleviated after FSHR knockdown (Figures 9a–d), suggesting that FSH-induced glutamate/GABA cycle imbalance can be mitigated by FSHR knockdown.

Taking together, FSH-induced neuroinflammation, hippocampal synaptic plasticity impairment and glutamate/GABA cycle disorders are mediated by FSHR in the hippocampus, and these pathologies can be mitigated by knockdown of FSHR.

### 3.5 FSH promotes inflammatory damage through the activation of the ERK pathway

**Figure 10:**
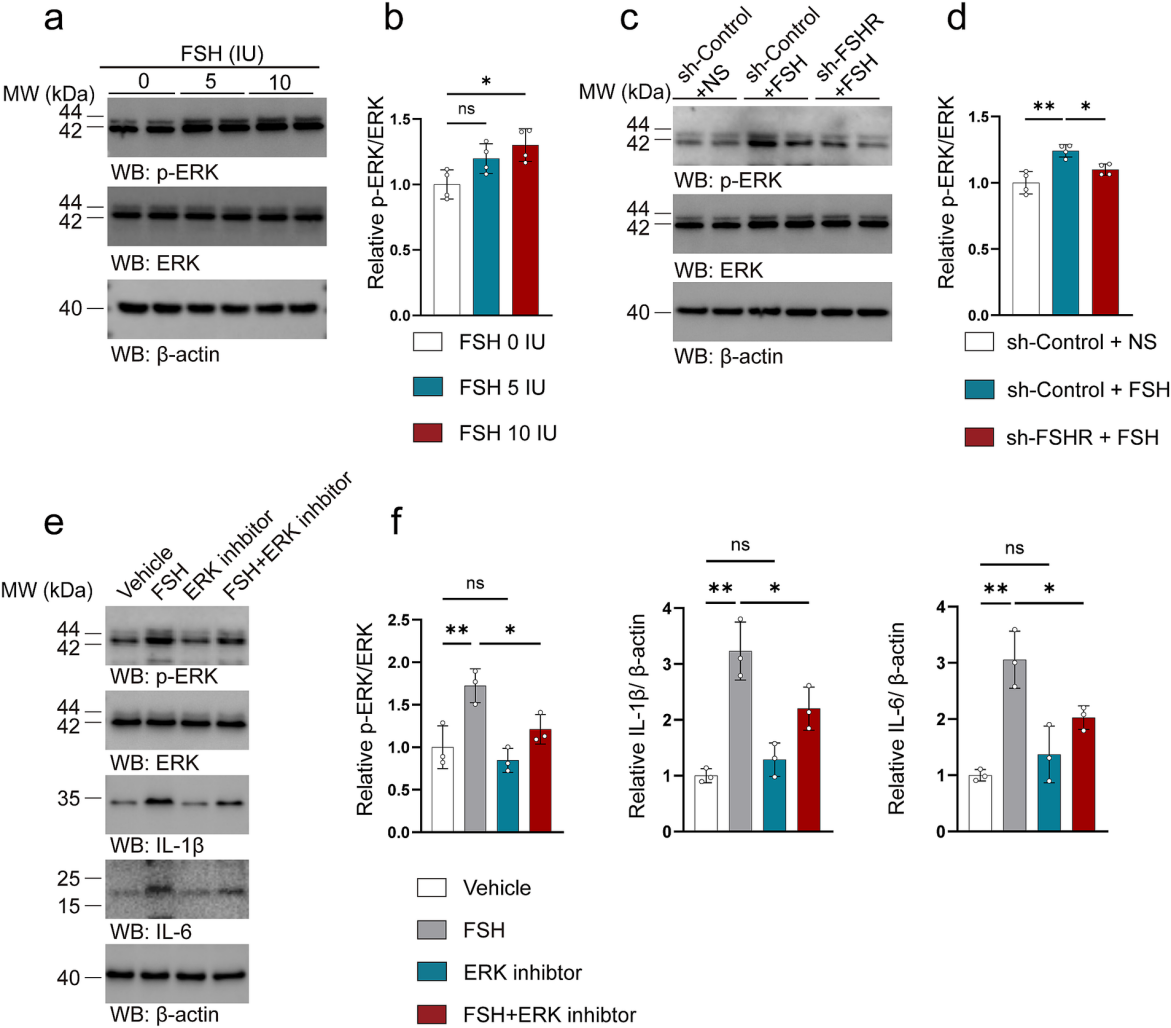
FSH promotes inflammatory damage through the activation of the ERK pathway. (a–d) Representative immunoblots images (a,c) and quantification (b,d) of ERK, p-ERK protein expression in the hippocampus, data are presented as mean ± SEM, *n* = 4 mice per group. In order to explore the machanism of FSH on neuroimflammtion, SH-SY5Y cells were assessed as the *in vitro* cell model, which were divided to the SY5Y cells into vehicle group, FSH group, ERK inhibitor group, and FSH + ERK inhibitor group. (e,f) Representative immunoblots images (e) and quantification (f) of ERK, p-ERK, IL-1β, IL-6 protein expression in SY5Y cells, data are shown as mean ± SEM of 3 independent experiments. Statistical analysis was performed by one-way ANOVA followed by Tukey’s multiple comparisons test. *p < 0.05, **p < 0.01; ns, not significant.

We attempted to explore the mechanism by which FSH contributes to the pathology associated with depression. Considering that our previous study found that FSH treatment directly phosphorylates ERK (Xiong et al., 2022) as well as another study mentioning that ERK plays a key role in regulating synaptic plasticity and the pathogenesis of depression (Zhuang et al., 2024). We investigated the expression and phosphorylation of ERK in the hippocampus of mice subjected to FSH treatment. Our findings corroborated previous cellular data, demonstrating a significant dose-dependent increase in ERK phosphorylation following FSH stimulation (Figures 10a,b). We also examined changes in ERK levels in the hippocampus after FSHR knockout, and the results showed that FSH-induced ERK phosphorylation were inhibited by FSHR knockdown (Figures 10c,d). We examined the correlation between ERK pathway activation and FSH-induced inflammation in SH-SY5Y cells. The results showed an upregulation in ERK phosphorylation following FSH stimulation, corroborating our *in vivo* results. Concurrently, it was observed that the FSH-induced increase in IL-1β and IL-6 levels was attenuated by the administration of an ERK inhibitor (Figures 10e,f). These results imply that the inflammatory damage induced by FSH is predominantly mediated through the activation of ERK phosphorylation, which is consistent with previous study (Mei et al., 2023).

## 4 Discussion

This study provides evidence that FSH induces anxiety and depression-like phenotype in a dose-dependent manner. We show that FSH binds to FSHR on the hippocampus and induces depressive behaviors, neuroinflammation, synaptic plasticity impairment, and neurotransmitter system imbalance in male mice. However, selectively downregulation of the FSH signaling in the hippocampus by FSHR knockdown may protect against FSH-induced depression. These results suggest that modulating the expression of FSH may be a potential method for the prevention of depression in females experiencing hormone fluctuation.

As previously noted, women are more likely to develop depression than men at various stages of their life, particularly in puberty, peripartum and menopause transition (Kuehner, 2017). The hormone fluctuation plays a vital role in depression. Recently, more and more studies showed that FSH, an important gonadotrophin, has been applied as a crucial hormone for the menopausal-related diseases. However, the role of FSH in depression is controversial among the clinical studies. In the present study, male C57BL mice were utilized to investigate the effects of FSH treatment, with the aim of mitigating the influence of hormonal fluctuations associated with the estrous cycle. We have found that the male mice exhibited depressive symptoms following a continuous four-week exposure to FSH. Specifically, FSH treated mice displayed increased immobility time in TST and FST, decreased sucrose preference in SPT, and less time and frequency in the center area in OFT. In addition, given that depression and anxiety are often comorbid with each other and have a significant overlap in pathophysiology (Chisholm et al., 2016; Mulder et al., 2019), we also examined anxiety-like behavior in the mice. The result reflects that FSH also induced anxiety-like behavior in mice in OFT and EMP test. Besides, both anxiety and depression can affect cognitive function, and most depressed patients may have memory deficits (Fisher et al., 2020). Therefore, we performed NOR test on FSH-treated mice to detect visual memory impairment, and our results showed that FSH induced cognitive impairment in these mice. This result is actually consistent with our previously published research showing that FSH acts directly on hippocampal and cortical neurons, impairs cognition in mice that exhibit features of Alzheimer’s disease (Xiong et al., 2022). Overall, our behavioral experiments demonstrated that FSH induced depression and anxiety phenotype in mice, and the behavioral tests on mice treated with FSH and FSHR knockdowns further confirm this conclusion.

Our findings are consistent with findings in the Harvard Study of Moods and Cycles, which found that FSH is positively related to depression in women before menopause. Analysis of data collected over a 36-month period from approximately 1,000 women indicated that FSH levels were consistently elevated in women with a history of depression compared to those without such a history (Harlow et al., 2003). Moreover, evidence from other studies shows that women with higher FSH levels were in greater risk of developing depression during post-partum (Ramachandran Pillai et al., 2017; Xi et al., 2023) Another research showed that increased FSH levels and increased FSH variability were significantly associated with the first onset of depression (Freeman, 2010). It is highly consistent with the results of another clinical study, which found that women whose FSH levels increased by 9 IU/L had more than two times the risk of depressive symptoms (Ryan et al., 2009). An animal study on rats also showed similar results, although the exact mechanism has not been elucidated, the study found that rats with high levels of FSH after ovariectomy were more likely to be depressed, and Danshen-Honghua could improve the stress-induced depression syndrome in stressed rats by regulating the level of FSH (Gu et al., 2018b). However, our findings do not align with the previous studies by Zhao He, who found that the FSHR ablation induced depressive behaviors, suggesting that FSH signaling is negatively correlated with depression (Bi et al., 2020a; Bi et al., 2020b). One possible explanation for this discrepancy is the differences in the animal models used in the studies. As shown in the previous study, the FSHR knock-out mice exhibit severe hypogonadism, characterized by irregular estrous cycles, ovulatory abnormalities, atrophic ovaries, and a thread-like uterus suggestive of pronounced estrogen deficiency (Sun et al., 2006; Dierich et al., 1998). The depressive symptoms observed in FSHR knockout mice are not solely attributed to FSHR inactivation but may also be influenced by estrogen insufficiency present since birth. However, specifically reducing FSHR expression in hippocampus neurons, which has a limited effect on the gonads and has no substantial impact on the other hormone levels, could alleviate the depressive symptoms induced by FSH. Therefore, our model illustrates that FSH can contribute to depression by binding to FSHR in the hippocampus.

Notably, emerging evidence indicates that neuroinflammation pathways are implicated in the pathogenesis of depression (Miller and Raison, 2016). Researches have demonstrated that pro-inflammatory cytokines have the potential to impair hippocampal-dependent memory and enhance depressive-like behaviors in murine models (Bourgognon and Cavanagh, 2020; Boitard et al., 2014). In addition, FSH has been found to cause endothelial inflammation (Wang et al., 2024). We examined pro-inflammatory cytokines in serum and hippocampus of FSH-treated mice and found elevated levels of IL-1β and IL-6 in hippocampus and IL-1β, IL-6, and TNF-*α* in serum. Our results are consistent with previous clinical studies, which showed that IL-1β, IL-6, and TNF-α levels were increased in patients with depression (Liu et al., 2024; Del Grande et al., 2016). Previous research has noted that increased levels of pro-inflammatory factors in the nervous system lead to the proliferation of neuroinflammatory cells, and the proliferation of neuroinflammatory cells will be further recruited to sites of injury or inflammation in the central nervous system (Malhi and Mann, 2018). Activation of microglia and astrocytes in the hippocampus is known to serve as indicators of inflammatory processes occurring in the brain. Meanwhile, microglia and astrocytes have been shown to be involved in the pathophysiology of various neuropsychiatric disorders (Kato et al., 2013). In our study, we found that FSH dose-dependently induced hippocampus microglial and astrocyte activation, and FSHR knockdown mitigated this phenomenon, which is in line with previous study (Xiong et al., 2023). Our results indicate FSH may trigger neuroinflammation and exacerbate depressive behaviors in male mice.

Since the synaptic plasticity impairment has been proven to be a vital pathological manifestation of depression (Duman et al., 2016; Duman and Aghajanian, 2012; Holmes et al., 2019), we tested the synaptic plasticity of FSH-treated mice by a variety of experimental techniques. Synapsin and PSD95 can be used as synaptic biomarkers to indirectly reflect synaptic plasticity (Yi et al., 2024), we found that the expression levels of synaptic proteins including PSD 95, synapsin and synaptophysin in hippocampus decreased after FSH treatment in a dose-dependent manner. Meanwhile, the results from Golgi staining and transmission microscopy in hippocampus provide additional evidence for FSH-induced synaptic plasticity damage.

Of particular note is that FSHR knockout in hippocampus could reduce FSH-induced synaptic plasticity impairment. To sum up, our study proved that FSH has a detrimental effect on synaptic plasticity.

The glutamic acid and GABA are excitatory and inhibitory neurotransmitters in the GABA transmission system, common research has long held that abnormalities in glutamate and GABA signaling are thought to play a role in depression (Lener et al., 2017; Garcia-Garcia et al., 2009; Draganov et al., 2020). The role of glutamate and GABA in depression has been reported in many articles. For instance, the findings of an independent study suggested that mice lacking GluR1 globally had impaired spatial working memory, accompanied by long-term memories of new environments (Weber et al., 2015). Another study demonstrated that GluR2/3 gene knockout mice exhibited hippocampal long-term depression (Meng et al., 2003). A study found that individuals with major depression had significantly lower levels of the glial excitatory amino acid transporters EAAT1 and EAAT2 in specific regions of the cerebral cortex (Choudary et al., 2005). Furthermore, another autopsy study had shown that in mood disorders, there is reduced expression of GAD65 and GAD67, the enzymes that convert glutamate into GABA (Fatemi et al., 2005). To gain a better understanding of the molecular mechanisms involved in FSH induced depressive behaviors, we conducted proteomics examinations of the mice brain after FSH treatment for 7 days. We found that the expression of proteins associated with glutamatergic synapse and glutamate receptor activity showed a significant difference in the brains of mice treated with or without FSH. Based on these findings, we examined the relevant indicators (VGluT1, GluR1 and GluR2) of glutaminergic system. We found that the expression levels of VGluT1 and GluR1 decreased after FSH treatment in a dose-dependent manner. Our results are consistent with the research mentioned above (Weber et al., 2015) and another research showing increased depression-like behavioral symptoms in VGluT1 heterozygote mice (Martisova et al., 2012). We also synchronously detected levels of a protein (GAD67) associated with the GABA energy system and found that both the fluorescence intensity and protein expression of GAD67 decreased in hippocampus of the mice treated with FSH. It is in line with the previous studies which showed that the dysfunction of GABA transmission system is associated with hormone fluctuation in ovarian-derived hormones and perimenopausal depression induced by hypothalamic–pituitary–adrenal (HPA) axis dysregulation (Gordon et al., 2015). Of particular note is that the FSH-induced disturbance of the glutamate /GABA cycle we mentioned above can be mitigated by FSHR knockout. In summary, our results indicated that FSH induces a disturbance in glutamatergic/GABAergic circuitry, potentially underlying the manifestation of depression-like behavior in mice.

Our findings demonstrate that follicle-stimulating hormone (FSH) can elicit depression-like behaviors in mice and provoke depression-associated pathological alterations. However, the underline mechanism by which FSH induces depression remains unclear. Previous research has identified the involvement of the ERK pathway in the hippocampus in the molecular pathophysiology of depression and emotion regulation. Additionally, another study has posited that elevated phosphorylation of hippocampal ERK1/2, mediated through enhanced activation of CRF1, may contribute to the depression-like behaviors observed in CRF2^−/−^ mice (Todorovic et al., 2009), which suggesting that ERK1/2 may serve as a critical target for investigating the neuronal mechanisms underlying stress-induced affective disorders (Tronson et al., 2008). Based on our hypothesis that FSH-induced neuroinflammation might be mediated by activation of the ERK pathway, we performed experiments in SH-SY5Y cells. Our findings indicated that the expression of inflammatory factors was increased in response to FSH treatment. However, co-treatment with an ERK inhibitor significantly mitigated this increase in inflammatory factor expression. These results suggest that the FSH-mediated elevation of inflammation levels is, at least in part, facilitated through the activation of the ERK pathway. These results were consistent with our previously findings (Xiong et al., 2022).

Taking together, this study has demonstrated that FSH could induced hippocampal neuroinflammation, synaptic plasticity impairment, GABA transmission system dysfunction and depressive behaviors in mice, which depends on the binding to FSHR in hippocampus. In effect, blocking FSH signaling is considered to be a novel way to treat a variety of diseases. For example, polyclonal antibodies (Ab) against the *β*-subunit of FSH has been found to prevent bone loss by inhibiting bone resorption and stimulating bone synthesis (Zhu et al., 2012; Ji et al., 2018). According to another study, this antibody sharply reduces adipose tissue in wild-type mice and also causes profound beiging, increases cellular mitochondrial density, activates brown adipose tissue and enhances thermogenesis (Liu et al., 2017). In addition, blocking FSH could alleviated the AD pathology in mice (Xiong et al., 2022). Targeting FSH may be a potential treatment for depression during hormone fluctuation.

## Data Availability

The raw data supporting the conclusions of this article will be made available by the authors, without undue reservation.

## Footnotes

^1^ https://www.omicshare.com/tools

## Supplementary material

SUPPLEMENTARY Figure S1 Proper dose of recombinant human FSH don’t interfere the serum level of 17β-estradiol and LH in mice. (a) ELISA quantification showing that human FSH levels in the serum of mice after recombinant human FSH treatment. (b,c) ELISA quantification shows that the17β-estradiol (b) and LH (c) levels in the serum of mice after recombinant human FSH treatment. Data are shown as mean ± SEM, *n* = 4–5 mice per group. Statistical analysis was performed by one-way ANOVA followed by Tukey’s multiple comparisons test. **p <* 0.05, ***p <* 0.01; ns, not significant.

SUPPLEMENTARY Figure S2 Intraperitoneal injection of NS did not increase the level of neuroinflammation in mice. (a,b) Representative immunoblots images (a) and quantification (b) of IL-1β protein expression in the hippocampus, data are presented as mean ± SEM, *n* = 4 mice per group. Statistical analysis was performed by unpaired two-tailed Student’s *t*-test, ns, not significant.

SUPPLEMENTARY Table S1 The folding changes and *p*-values of significantly altered protein (*P**, 0 IU group vs. 5 IU group; *P*^#^, 0 IU group vs. 10 IU group).

SUPPLEMENTARY Table S2 The folding changes and *p*-values of significantly altered protein (*P**, sh-Control + NS group vs. sh-Control + FSH group; *P*^#^, sh-Control + NS group vs. sh-FSHR + FSH group).

SUPPLEMENTARY Table S3 The folding changes and *p*-values of significantly altered protein (*P**, vehicle group vs. FSH group; *P*^#^, vehicle group vs. ERK inhibitor group; *P*^##^, vehicle group vs. FSH + ERK inhibitor group).
