# Supplementary material for: Follicle-stimulating hormone induces depression-like phenotype by affecting synaptic function
Source: Front Mol Neurosci. 2024 Oct 21;17:1459858. doi: 10.3389/fnmol.2024.1459858 (PMC11532131; doi:10.3389/fnmol.2024.1459858)
Supplement: Supplementary file 1 [file Data_Sheet_1.pdf]

Supplementary materials for

Follicle-Stimulating Hormone Induces Depression-Like Phenotype by Affecting Synaptic Function

Liqin Huang, Shangqi Sun, Gege Jiang, Guanfeng Xie, Yunying Yang, Sichun Chen, Jiaying Luo, Chen Lv, Xiang Li, Jianming Liao, Zhihao Wang, Zhaohui Zhang, Jing Xiong

Supplementary figures

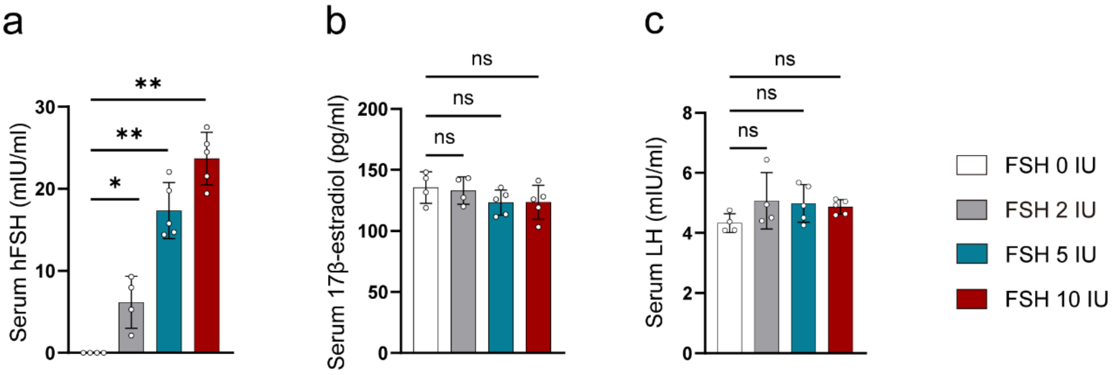

Supplementary Figure 1 Proper dose of recombinant human FSH don't interfere the serum level of 17β-estradiol and LH in mice.

(a) ELISA quantification showing that human FSH levels in the serum of mice after recombinant human FSH treatment. (b, c) ELISA quantification shows that the17β-estradiol (b) and LH (c) levels in the serum of mice after recombinant human FSH treatment. Data are shown as mean ± SEM, n = 4-5 mice per group. Statistical analysis was performed by one-way ANOVA followed by Tukey's multiple comparisons test. $*P < 0.05$, $**P < 0.01$; ns, not significant.

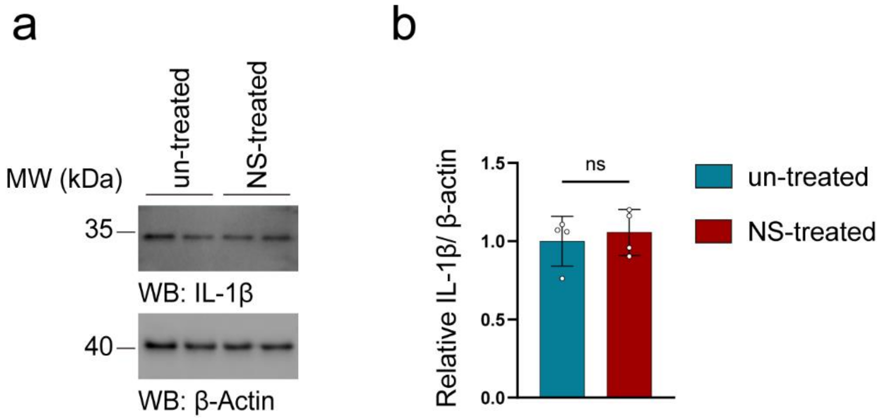

Supplementary Figure 2 Intraperitoneal injection of NS did not increase the level of neuroinflammation in mice.

(a-b) Representative immunoblots images (a) and quantification (b) of IL-1β protein expression in the hippocampus, data are presented as mean ± SEM, n = 4 mice per group. Statistical analysis was performed by unpaired two-tailed Student's t-test, ns, not significant.

Supplementary Table 1

	protein	0IU		5IU		10IU	
		Fold	*P*	Fold	*P* *	Fold	*P* #
Fig. 2b	IL-1B	1		1.682	0.0017	2.38	<0.0001
	IL-6	1		1.872	0.0522	2.515	0.0021
Fig. 3b	PSD95	1		0.8011	0.0412	0.5512	0.0002
	Synapsin	1		0.8165	0.0065	0.4398	<0.0001
	Synaptophysin	1		0.9238	0.5937	0.6919	0.0073
Fig. 4b	GluR1	1		0.7092	0.0113	0.4515	0.0001
	GluR2	1		1.143	0.0551	1.164	0.4272
	VGlut1	1		0.8979	0.6021	0.5831	0.0027
	GAD67	1		0.7193	0.0207	0.3507	<0.0001
Fig. 10b	p-ERK	1		1.198	0.1163	1.301	0.016

Supplementary Table 1

The folding changes and P-values of significantly altered protein (*P**, 0 IU group vs. 5 IU group; $P^{\#}$, 0 IU group vs.10 IU group).

Supplementary Table 2

	protein	sh-Con+NS		sh-Con+FSH		sh-FSHR+FSH	
		Fold	*P*	Fold	*P**	Fold	$P^{\#}$
Fig. 5d	FSHR	1		0.3857	0.0131		
Fig. 7b	IL-1β	1		2.809	<0.0001	1.731	0.0004
	IL-6	1		2.078	0.0006	1.332	0.0066
Fig. 8b	PSD95	1		0.5393	0.0002	0.9092	0.0008
	Synapsin	1		0.3915	<0.0001	0.7955	0.0018
	Synaptophysin	1		0.6794	0.0112	0.9203	0.0454
Fig. 9b	GluR1	1		0.6374	0.0003	0.8394	0.0124
	GluR2	1		0.9846	0.01543	0.9181	0.06648
	VGlut1	1		0.7038	0.0041	0.8926	0.0464
	GAD67	1		0.5766	0.0004	0.9735	0.0007
Fig. 10d	p-ERK	1		1.241	0.0006	1.1	0.017

Supplementary Table 2

The folding changes and P-values of significantly altered protein (*P**, sh-Control + NS group vs. sh-Control + FSH group; $P^{\#}$, sh-Control + NS group vs. sh-FSHR + FSH group).

Supplementary Table 3

	protein	Vehicle		FSH		inhibitor		FSH+inhibitor	
		Fold	*P*	Fold	*P* *	Fold	*P* #	Fold	*P* ##
Fig. 10f	P-ERK	1		1.723	0.0083	0.8461	0.7729	1.211	0.5772
	IL-1β	1		3.232	0.0004	1.288	0.9298	2.2	0.0214
	IL-6	1		3.058	0.0007	1.37	0.6413	2.025	0.0416

Supplementary Table 3

The folding changes and P-values of significantly altered protein (P*, vehicle group vs. FSH group; $P^{\#}$, vehicle group vs. ERK inhibitor group; $P^{\#\#}$, vehicle group vs. FSH + ERK inhibitor group).
